# Research progress on the forkhead box C1

**DOI:** 10.18632/oncotarget.22527

**Published:** 2017-11-20

**Authors:** Jinhua Wang, Wan Li, Xiangjin Zheng, Xiaocong Pang, Guanhua Du

**Affiliations:** ^1^ The State Key Laboratory of Bioactive Substance and Function of Natural Medicines, Beijing 100050, China; ^2^ Key Laboratory of Drug Target Research and Drug Screen, Institute of Materia Medica, Chinese Academy of Medical Science and Peking Union Medical College, Beijing 100050, China

**Keywords:** FOX family, FOXC1, cancer, drug resistance, stem cell

## Abstract

FOXC1 is a vital member of FOX families which play important roles in biological processes including proliferation, differentiation, apoptosis, migration, invasion, metabolism, and longevity. Here we are focusing on roles of FOXC1 and their mechanisms in cancers. FOXC1 promoted progress of many cancers, such as breast cancer (especially basal-like breast cancer), hepatocellular carcinoma, gastric cancer and so on. FOXC1 was also found to be associated with drug resistance of cancers. FOXC1 promoted metastasis of cancers by increasing expression of MMP7, NEDD9 and Snail. Proliferation and invasion of cancers were increased by FOXC1 by mediating NF-κB, MST1R and KLF4 expression. FOXC1 was associated with development by regulating expression of FGF19 and MSX1. Recently, FOXC1 was found to be required for niche of stem cells or development of stem cells by mediating expression of Gli2, CXCL12, SCF, NFATC1, BMP and Myh7. Overall, FOXC1 exerts its functions by many mechanisms and may be used as a potential biomarker for diseases.

## INTRODUCTION

### FOX family

Forkhead box (FOX) proteins are describled with a common DNA-binding forkhead domain. FOX domain is a ∼100-amino-acid conserved forkhead domain which can bind DNA. FOX proteins which are a superfamily of evolutionarily conserved transcription factors play many important roles in biological processes including development, differentiation, proliferation, apoptosis, metabolism, migration, invasion and longevity [1–3]. Currently, human FOX gene family consists of 49 members including FOXA1-FOXS1. These forty-nine FOX members are categorized into 19 subfamilies (FOXA-FOXS) [4].

### History of FOXC1 and axenfeld-rieger syndrome

Axenfeld-Rieger syndrome (ARS) is an eye disease which has an autosomal dominant genetic changes including systemic abnormalities and anterior segment dysgenesis. The eye disease was identified by Axenfeld and named as Axenfeld anomaly in 1920. Patients with congenital iris abnormalities including polycoria, iris hypoplasia and correctopia were described and named by Rieger in 1935. The combination of Reiger syndrome and Axenfeld anomaly is named as ARS.

FOXC1 (Forkhead Box C1) is also named as FKHL7. FOXC1 codes 553 amino acids and its molecular weight is 57KD. FOXC1 was firstly found to be associated with Axenfeld-Rieger syndrome and harbor mutations in patients which were diagnosed as Rieger anomaly (RA), Iris Hypoplasia (IH) and Axenfeld anomaly (AA) [5]. Mutations in FOXC1 were demonstrated to lead to many glaucoma phenotypes [6, 7]. More and more studies showed that there are new mutations in FOXC1, PITX2 and PAX6 in families which have anterior segment dysgenesis disorders [8, 9], that there were mutations in the wing region of the FOXC1 gene forkhead domain [10–15].

## FOXC1 AND CANCERS

### Functions of FOXC1 in cancers

#### FOXC1 and breast cancer

FOXC1 was firstly reported to be overexpressed in basal-like breast cancer (BLBC) by our group. Elevated FOXC1 expression was associated with poor prognosis of BLBC patients. Ectopic FOXC1 expression promoted cell migration, invasion and proliferation, whereas knockdown of FOXC1 expression induced opposite effects in breast cancer cells [16]. We further confirmed that FOXC1 exerted its functions in BLBC cells by activating NF-κB signaling [17]. FOXC1 expression by immunohistochemistry detection in triple negative invasive breast cancer is an independent biomarker of survival rate in BLBC patients [18]. Expression of FOXC1 was dramatically correlated with expression of EGFR in human breast cancers. FOXC1 expression was induced by EGFR in BLBC while inhibition of EGFR led to decrease of FOXC1 expression in xenograft tumors. Knockdown of FOXC1 inhibited the effects of EGF on migration, invasion and proliferation of BLBC cell [19]. Expression of FOXC1 was correlated with Gli2 expression and its downstream targets in breast cancers. In addition, overexpression of FOXC1 significantly decreases sensitivity to Hedgehog (Hh) inhibitors in BLBC cells as well as xenograft tumors. FOXC1 activates smoothened-independent Hh signal pathway in BLBC [20]. Combination of FOXC1 expression with matrix metalloprotease 7 (MMP7) expression can be used as an independent predictor of patient outcome in multivariate analyses of two breast cancer patient cohorts. FOXC1 promotes metastasis and invasion by inducing MMP7 expression in breast cancer [21]. GATA3 and BRCA1 were found to inhibit the development of BLBCs by inhibiting expression of FOXC1. Abnormal FOXC1 expression promotes the epithelial-to-mesenchymal transition (EMT)-like phenotypes and drug resistance which contributed to aggressive BLBCs [22]. Recently, we found that FOXC1 expression was closely associated with ERα expression. FOXC1 overexpression reduced ERα expression while knockdown of FOXC1 increase ERα expression. FOXC1 expression inhibited responses to estradiol (E2) and Tamoxifen in the MCF-7. In addition, FOXC1 expression reduced expression of ERα downstream targets including progesterone receptor and XBP1, and dramaticlly inhibited ERE luciferase activity caused by E2 [23].

#### FOXC1 and liver cancer

Expression of FOXC1 was significantly higher in Hepatocellular carcinoma (HCC) tissues than that in corresponding normal tissues. HCC patients with low FOXC1 expression had longer overall survival times and lower recurrence rates than those with high FOXC1 expression. FOXC1 expression was an independent, important factor for survival and recurrence after operation. FOXC1 overexpression led to significant changes of EMT and improvement of invasion in HCC cell and lung metastasis while knockdown of FOXC1 reduced these processes. FOXC1 induced expression of Snai1, but inversely reduced E-cadherin expression in human HCC tissues [24]. FOXC1 was showed to increase microvascular invasion of primary HCC via regulating EMT [25]. It was reported that IL-8 activates expression of FOXC1 via PI3K/AKT pathway and hypoxia-inducible factor 1α, and that FOXC1 expression induced transactivation of CXCR1 and CCL2, and promoted inflammation, migration and invasion of HCC cells [26].

#### FOXC1 and lung cancer

Both FOXC1 mRNA expression and protein expression in the tissues of NSCLC were dramatically higher than those in adjacent normal tissues. Overexpression of FOXC1 was related to poor prognosis of tumor, stage of tumor node metastasis, and lymph node metastasis. Patients with low FOXC1 expression had higher overall survival rate than those with high expression. According to clinical statistics, FOXC1 expression could be used as an independent prognostic biomarker in NSCLC patients [27]. Silencing FOXC1 may significantly inhibit the migration of lung cancer cells by changing the EMT process through inhibiting expression of cadherin, being associated with the expressions of extracellular MMPs [28].

#### FOXC1 and leukemia

FOXC1 was expressed in more than 20% of human Acute Myeloid Leukemia (AML) patients while there was no expression of FOXC1 in normal hematopoietic populations. FOXC1 expression was significantly correlated with expression of the HOXA/B locus in AML. FOXC1 inhibits differentiation of monocyte/macrophage and increases clonogenic potential. *In vivo* analyses, FOXC1 dramatically promoted the onset of symptomatic leukemia with HOXA9. Human AML cases who have high FOXC1 expression showed little morphologic monocytic differentiation and inferior survival [29].

#### FOXC1 and other cancers

FOXC1 overexpression caused by hypomethylation of FOXC1 gene promoted proliferation, migration and invasion of melanoma cells. FOXC1 exerted functional roles by activating the MST1R/PI3K/AKT pathway in melanoma. FOXC1 expression can be used as a biomarker in melanoma patient [30].

mRNA and protein expression of FOXC1 in gastric cancer (GC) tissues were dramatically higher than those in adjacent normal tissues. Elevated FOXC1 expression was correlated with the degree of histological differentiation, lymph node metastasis, distant metastasis, TNM stage and invasive depth. Survival analysis showed that patients with low FOXC1 expression had higher overall survival rate than those with high expression. Clinical statistical analysis demonstrated that overexpression of FOXC1 could be used as an independent prognostic biomarker in GC patients [31].

FOXC1 mRNA and protein was highly expressed in pancreatic ductal adenocarcinoma (PDA) tissues compared with corresponding normal tissues. Elevated FOXC1 expression was correlated with the degree of histological differentiation, lymph node metastasis, distant metastasis, TNM stage and invasive depth. Survival analysis demonstrated that overexpression of FOXC1 is closely correlated with a poor prognosis [32].

Esophageal squamous cell carcinoma (ESCC) patients with low FOXC1 expression have a significantly better prognosis than those with high FOXC1 expression. FOXC1 significantly increased cell proliferation, colony formation, migration and invasion in ESCC cells [33].

Upregulation of FOXC1 was associated with progression of prostate cancer [34].

### FOXC1 and resistance of cancer drug

FOXC1 plays important roles in and is involved in development of many kinds of stem cells. FOXC1 promoted progress of cancers by activating many signal pathways. More and more study showed that cancer stem cells (CSCs) which were associated with metastasis, relapse of cancers and drug resistance could dramatically influence treatment of tumor. Overexpression of FOXC1 led to resistance of melanoma cells to BRAF inhibitor PLX4032 [30]. MCF-7-FOXC1 cells with high FOXC1 expression are less sensitive to Tamoxifen than MCF-7 control cells without FOXC1 expression [23]. Overexpression FOXC1 led to resistance of Hh inhibitors in BLBC and xenograft tumors [20]. Recently, our data showed that overexpression of FOXC1 increased resistance to Doxorubicin or Paclitaxel in triple negative breast cancer cells and tumors (Data wasn’t published now).

## FOXC1 AND OTHER DISEASES

FOXC1 is involved in many cellular processes including development, proliferation, differentiation, cell cycle, migration and invasion, metabolism and DNA damage response of cells.

### Stem cell

FOXC1 is involved in development of many stem cells. In the embryo, it was well studied that progenitors of vascular smooth muscle cells (SMCs) which are originated from multiple types of progenitor cells are the cardiac neural crest stem cells. Differentiation of Smooth muscle in the neural crest lineage is mediated by FOXC1 [35]. The periocular mesenchyme developed from neural crest (NC) stem cell partly promoted development of eye. TGF-β2 from the lens promotes expression of Pitx2 and FOXC1 in NC-derived cornea [36]. Bone marrow provides special microenvironments for both haematopoietic stem and progenitor cells. FOXC1, known as a specific transcriptional regulator, play important roles in maintaining development of haematopoietic stem and progenitor cells [37]. Many BLBC cells which have CSC properties were activated through Smoothened (SMO)-independent Hh signal pathway by Gli2 [20]. FOXC1-deficient hair follicle stem cells (HFSCs) spend less time in quiescence, which led to significantly shorten resting periods between hair cycles. FOXC1 sustains niche of the HFSCs and regulated stem cell quiescence to preserve long-term tissue-regenerating potential [38]. In self-renewing hair follicle stem cells, both nuclear factor of activated T-cells 1(NFATC1) and bone morphogenetic protein (BMP) signal pathways were active by FOXC1. FOXC1 plays important role in strengthening quiescence in maintaining hair follicle stem cells [39]. FOXC1 regulated the early stage of cardiomyogenesis by many pathways such as calcium signaling, tight and gap junctions, cell adhesion, and actin cytoskeleton. Myh7 is expressed during cardiac development and is a downstream target of FOXC1. In addition, FOXC1 mediated the functional properties of cardiomyocytes which are derived from embryonic stem cell (ESC) [40]. Bingchen Han et al reported that expression of FOXC1 was correlated with Gli2 expression and its downstream targets in breast cancers, and that FOXC1 maintained CSC properties in BLBC cells *in Vivo* and *in Vitro* by mediating non-canonica Gli2-Hh signal pathway [20].

### FOXC1 and other diseases

It was reported that mutations in the FOXC1 gene caused defects in the anterior segment of the eye, mitral valve defects and atrial septal defects [41]. FOXC1 was found to play an important role in the morphogenesis of the cardiovascular system [42]. Both FOXC1 and FOXC2 are need for the morphogenesis of the cardiac outflow tract [43]. FOXC1 overexpression increased expression of Notch1 and its corresponding ligand Delta-like 4 (Dll4), which are known as arterial markers. Both FOXC1 and FOXC2 can bind to Dll4 promoter via a Foxc-binding site. In addition, mutation of FOXC1 inhibited the initial development of lymphatic endothelial cells [44]. The early organogenesis of the kidney and urinary tract was promoted by FOXC1 [45, 46].

Many studies show that FOXC1 is an important component of the signal pathways which mediates embryonic heart development and cardiogenesis, playing key important roles in development of heart [47]. Both mutations in FOXC1 and the abnormal expression of FOXC1 significantly affect the congenital heart disease (CHD) of humans [48].

## MECHANISMS OF FOXC1

**Figure 1:**
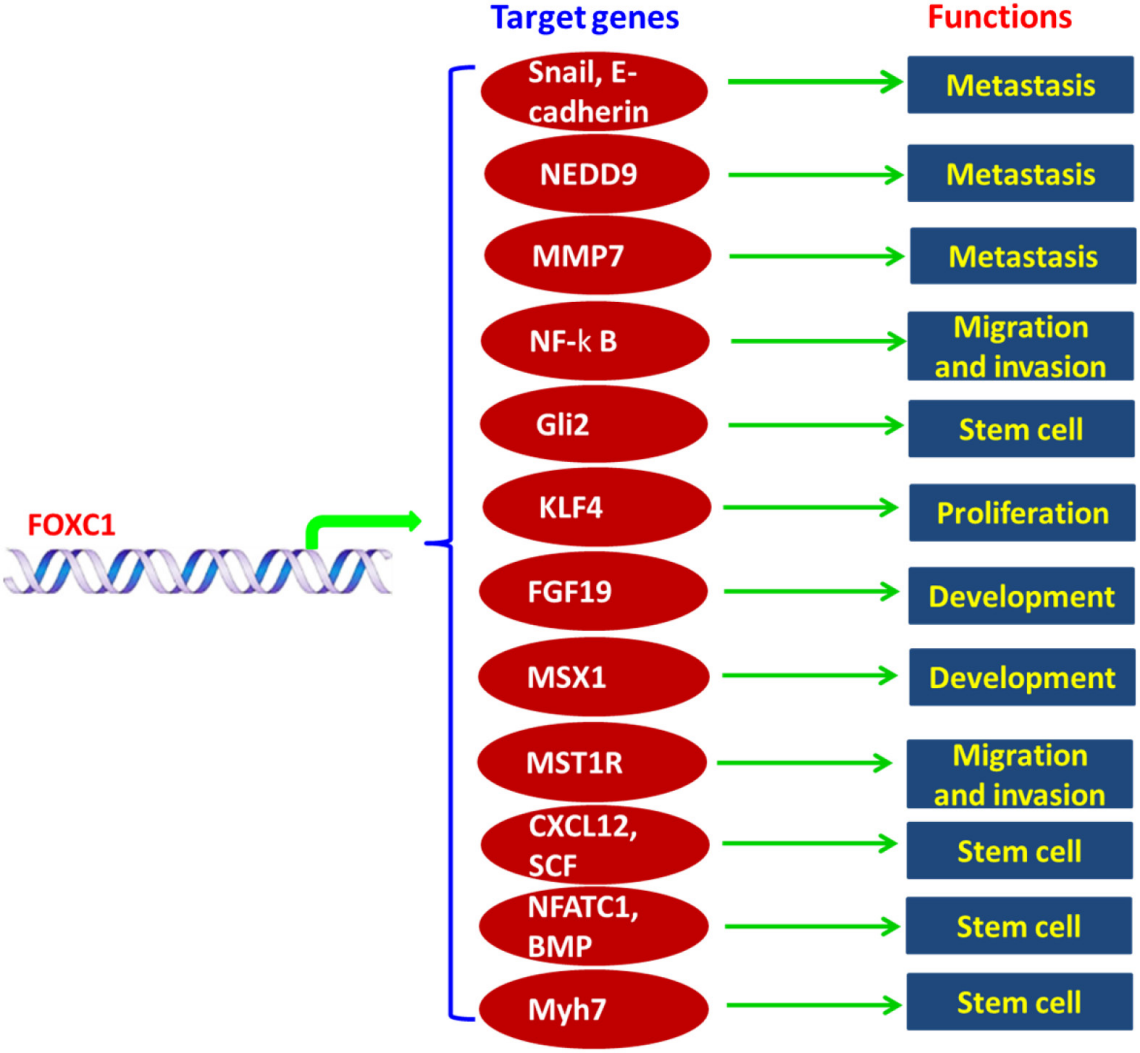
Functions and mechanisms of FOXC1 in human

FOXC1 plays complex and important roles in biological processes from development and organogenesis. FOXC1 exerts many functions by different mechanisms (Figure 1).

### FOXC1 induced snai1 expression and promoted epithelial-to-mesenchymal transition

FOXC1 induced expression of Snai1, but inversely reduced E-cadherin expression in human HCC tissues. FOXC1 increased expression of Snail by directly binding to its promoter, thereby inhibited expression of E-cadherin and promoted EMT [24, 25].

### FOXC1 induced neural precursor cell expressed developmentally down-regulated 9 (NEDD9) expression and promoted FOXC1 invasion and metastasis

Compelling evidence, such as DNA microarray, site directed mutagenesis et al, confirmed that NEDD9 is a downstream target of FOXC1. FOXC1 upregulated the expression of NEDD9 and promoted invasion and metastasis of HCC [24].

### FOXC1 induced MMP7 expression and promotes invasion of breast cancer

FOXC1 expression is significantly associated with expression of MMP7 in breast cancer samples and cell lines at both the mRNA and protein levels. Ectopic overexpression of FOXC1 in nontransformed mammary epithelial cell lines significantly increased expression of MMP7. On the contrary, knockdown of FOXC1 inhibited expression of MMP7 without affecting expression of other matrix metalloproteinases in BLBC cell lines [21].

### FOXC1 activated NF-κB pathway

FOXC1 inhibited P65 degradation by increasing expression of PIN1 (a peptidyl-prolyl isomerase) and reducing expression of SOCS1 (suppressor of cytokine signaling 1) in triple negative breast cancer cells. FOXC1 overexpression caused more sensitive to pharmacological inhibition of NF-κB breast cancers. FOXC1 promoted cell proliferation, migration and invasion by activating NF-κB pathway [17].

### FOXC1 activated smoothened-independent hedgehog signal pathway

Expression of FOXC1 was significantly correlated with that of Gli2 and its downstream targets in breast cancers. Many BLBC cells which were activated by FOXC1 maintained CSC properties via activation of Smoothened (SMO)-independent Hh signaling by Gli2. FOXC1 directly binds to the promoter of Gli2 and regulated Gli2 expression. Overexpression of FOXC1 caused more resistance to Hh inhibitors in BLBC cells and xenograft tumors. [20].

### FOXC1 reduced expression of KLF4 and collaborated with HOXA9

FOXC1 inhibited differentiation of monocyte/macrophage and enhanced clonogenic potential by reducing expression of KLF4. *In vivo* analyses, FOXC1 significantly accelerated the onset of symptomatic leukemia by collaborating with HOXA9 [29].

### FOXC1 activated the FGF19–FGFR4-MAPK pathway

FOXC1 positively mediates expression of FGF19 in both corneal and periocular mesenchymal cells which are from zebrafish embryos or cultured cells. FGF19 was confirmed as a downstream target of FOXC1 in the eye. FGF19 increases MAPK phosphorylation by the FGFR4 tyrosine kinase in the both developing and mature cornea. Knockout of FOXC1 expression or FGF19 expression caused complementary, but distinct, anterior segment dysgeneses. FOXC1 plays an important role in both the development and maintenance of anterior segment structures within the eye by activating FGF19–FGFR4-MAPK pathway [49].

### FOXC1 activated MSX1- NKX3-1 pathway

FOXC1 mediated activation of MSX1 transcription. Overexpression of FOXC1 induced upregulation of MSX1. Overexpression of the both transcriptional factor GATA3 and TAL1 significantly decreased expression level of MSX1 in PER-117 cells while knockdown of GATA3 and TAL1 markedly induced expression of MSX1 in JURKAT cells. Knockdown of MSX1 reduced expression of NKX3-1, which demonstrated that MSX1 activated NKX3-1 as well. MSX1 regulated the NKL homeobox gene NKX3-1 which may play an important role in T-cell acute lymphoblastic leukemia(T-ALL)[50].

### MST1R/PI3K/AKT pathway was activated by FOXC1

FOXC1 play an important role in melanoma cells, such as invasion, migration and proliferation, colony formation and growth in 3D Matrigel. Ectopic FOXC1 expression induced MST1R expression and promoted migration and invasion of melanoma cells by activating the MST1R/PI3K/AKT pathway. Inhibition of PI3K/AKT significantly reduced migration and invasion mediated by FOXC1 [30].

### FOXC1 regulated SCF and CXCL12 expression

FOXC1 is found to be expressed in the CXC chemokine ligand (CXCL) 12-abundant reticular (CAR) cells which are necessary for maintenance of haematopoietic stem and progenitor cell *in vivo* in the developing and adult bone marrow. Haematopoietic stem and progenitor cells were significantly reduced if FOXC1 was knock out in CAR cells or marrow mesenchymal cells. FOXC1 upregulates expression of CXCL12 and stem cell factor (SCF) and promotes development of CAR cells. FOXC1 plays an important role in maintaining the mesenchymal niches for haematopoietic stem and progenitor cells [37].

### FOXC1 activated Nfatc1 and bone morphogenetic protein (BMP) signaling

Expression of FOXC1 is increased when murine hair follicle stem cells (SCs) is activated. SCs can’t reestablish quiescence and allow premature activation if FOXC1 is deleted in activated SCs. The loss of old hair was caused by knockdown of FOXC1 in the SC. FOXC1 mediates BMP and Nfatc1 signal pathways to govern quiescence in self-renewing SCs [39].

### FOXC1 regulated expression of Myh7 during cardiac development

FOXC1 regulated many pathways which are implicated in cardiac functions, such as calcium signaling, actin cytoskeleton, gap and tight junctions, and cell adhesion. Expression of Myh7 is regulated by FOXC1 during cardiac development. FOXC1 mediated the functional properties of cardiomyocytes which are derived from ESC [40].

## CONCLUSIONS AND FUTURE PERSPECTIVES

Up to now, it is unclear how FOXC1 expression is regulated. Some studies showed that epigenetic changes of FOXC1 were involved in FOXC1 expression. It is very important to study FOXC1 expression regulated by epigenetics and elucidate the mechanism of FOXC1 expression in the future. Study and RNA-Seq data showed that expression FOXC1 was involved with inflammation. More studies will be focused on the effects of FOXC1 on inflammation in cancer microenvironment. More importantly, there is no inhibitor of FOXC1. It is necessary to establish high throughput screen model for screening FOXC1 inhibitor and develop FOXC1 inhibitor as an anti-cancer drug.

FOXC1, an important member of FOX family, is highly expressed and plays critical roles in many cancers and stem cells. FOXC1 can be used as a potential biomarker of diseases.
